# Development of Ti–Al–V alloys for usage as single-axis knee prostheses: evaluation of mechanical, corrosion, and tribocorrosion behaviors

**DOI:** 10.1038/s41598-023-31548-1

**Published:** 2023-03-16

**Authors:** B. O. Pinto, J. E. Torrento, C. R. Grandini, E. L. Galindo, C. A. F. Pintão, A. A. Santos, P. N. Lisboa-Filho, F. M. L. Pontes, D. R. N. Correa

**Affiliations:** 1 Laboratório de Anelasticidade e Biomateriais, São Paulo State University (UNESP), School of Sciences, Bauru, SP 17033-360 Brazil; 2 Laboratório de Caraterização Física e Reológica, São Paulo State University (UNESP), School of Sciences, Bauru, SP 17033-360 Brazil; 3 Laboratório de Nanotecnologia e Materiais Avançados, São Paulo State University (UNESP), School of Sciences, Bauru, SP 17033-360 Brazil; 4 Departamento de Química Faculdade de Ciências, São Paulo State University (UNESP), School of Sciences, Bauru, SP 17033-360 Brazil

**Keywords:** Biomaterials, Materials for devices

## Abstract

Single-axis knee prosthesis is an artificial biomechanical device that provides motion to amputees without the need for assistance appliances. Besides it is mainly composed of metallic materials, the current commercial materials did not group adequate properties for long-term usage or accessible cost. This study produced and characterized Ti-(10 −x)Al-xV (x = 0, 2, and 4 wt.%) alloys for potential use as single-axis knee prostheses. The samples exhibited a gradual decrease in the density values, with proper chemical mixing of the alloying elements on the micro-scale. The phase composition exhibited a primary α phase with a minor α′ + β phase for the Ti-8Al-2V and Ti-6Al-4V samples. Due to their different atomic radius compared to Ti, the addition of alloying elements changed the cell parameters. Their selected mechanical properties (Young’s modulus, Vickers microhardness, and damping factor) performed better values than the CP-Ti grade 4. The samples also exhibited good corrosion properties against the simulated marine solution. The tribocorrosion resistance of the samples was better than the reference material, with the wear tracks composed of some tribolayers and grooves resulting from adhesive and abrasive wear. The Ti-10Al alloy displayed the best properties and estimated low cost to be used as single-axis knee prostheses.

## Introduction

Knee prosthesis is a biomechanical device used by leg amputee patients and is an efficient way to recover from walking, jogging, and standing without needing assistive appliances. Leg amputation results from injuries (traffic or workplace accidents) or diseases (chronic or age-related) requiring the limb's surgical removal^1^. The prosthetic knees can be mechanical or computerized, having the first one the cheaper choice for the patient. The mechanical knee prosthesis is also divided according to the number of axes of rotation, which can be single-axis, multi-axis, or polycentric^2^. The single-axis is the simplest type which permits only flexion and extension of the knee, being the best cost-effective option for poor and older people^2,3^. The main disadvantages evolve the excessive muscle power needed to keep a stable walking and standing, and the difficulty in controlling knee rotation, which could impact the gait pattern and produce a risk of falls and injuries^4^. Considering that the current stainless steel prostheses do not meet the clinical needs, the design of low-cost Ti-based alloys can potentially surpass these drawbacks without excessively increasing costs.

Ti and its alloys have mainly been employed as biomedical materials due to their favorable mechanical, corrosion, wear, and biological properties. The usage mainly consists of implants and devices for orthopedy, cardiology, and odontology^5^. The combination of alloying elements and proper thermo-mechanical treatments can change the proportion of α (hexagonal close-packed crystalline structure, hcp) and β phase (body-centered cubic crystalline structure, bcc) or precipitate metastable phases (such as martensitic α′ and α″ or ω) which can impact the Ti properties directly^6^. The formation of Ti solid solutions with non-toxic alloying elements has been currently established as a smart strategy to overcome the limitations regarding the stress shielding effect promoted by Young’s modulus mismatch with the human bone, failures promoted by the corrosion from body fluids, and toxicity resulted from released ions and debris originated by wear mechanisms^7^. However, developing novel Ti-based materials that meet all clinical needs is still challenging.

Ti-6Al-4V alloy, also called CP-Ti grade 5, is designated by the ASTM F136 standard^8^ and is currently the most popular Ti alloy worldwide. The material was developed in the 1950s targeting the usage in structural components of aircraft and airspaces due to its lightweight, high strength, excellent fracture toughness, and good corrosion resistance provided by its dual α + β phase composition. In the 1970s, the material began to be used as a biomaterial, specifically in manufacturing orthopedical implants. However, some concerns about the ion-releasing of harmful and toxic Al and V ions are still warning the Medicine^9,10^. In this scenery, some strategies for overcoming this drawback and open novel applications are based on adding alloying elements, such as Ti–Al–V–X (X = Fe, Zr, and Mo) alloys^11–13^. However, the current studies only focused on the potential applications of Ti alloys in biomedical implants without considering external prostheses, despite the extensive demands in the field.

From these previous considerations, developing a low-cost Ti–Al-based alloy could be an interesting approach to be used by amputee people. In this scenery, this paper aims to produce and characterize Ti-(10−x)Al-xV (x = 0, 2, and 4 wt%) alloys for potential use as single-axis knee prostheses for the first time. The samples were characterized by chemical and phase composition, density, structure, microstructure, and selected mechanical, electrochemical, and tribocorrosion properties. Their processing cost was estimated based on the pricing of the raw materials.

## Materials and methods

Ti-(10−x)Al-xV (x = 0, 2, and 4 wt.%) samples were produced from commercially pure Ti (CP-Ti grade 2)^14^, pure Al, and Ti-6Al-4V (CP-Ti grade 5)^8^ after cleaning in aqueous ultrasonic bath and separation in the corresponding mass proportion. The ingots were cast in an argon arc-melting furnace with a water-cooled copper crucible and tungsten electrode. The chamber was previously cleaned in a vacuum of 10^–3^ Torr and later purged with argon gas until 10^2^ Torr. The samples were re-melted five times to ensure adequate chemical mixing. Then, the samples were submitted to a homogenization heat treatment at a vacuum of 10^–5^ Torr, a heating rate of 10 K min^−1^, a plateau of 1273 K, for 21.6 ks, and the furnace cooled. Later, the samples were hot-rolled at 1273 K, with a thickness reduction of around 5 mm, and air-cooled. Finally, the samples were solutionized at 10^–5^ Torr, in 1173 K, for 7.2 ks, and water quenched for stress relief and microstructural recrystallization.

In the semi-quantitative chemical analysis and elemental mapping mode, the chemical composition was assessed by X-ray dispersive spectroscopy (EDS; Inca X-Act detector, Oxford Inc.). Density values were acquired using Archimedes’ principle and a digital balance (0.0001 g) at room temperature and compared to the theoretical values obtained from the weighted average of the alloying elements. The phase composition was evaluated by X-ray diffraction (XRD; Rigaku diffractometer, MiniFlex 600 model) at 40 kV and 15 mA, Ni-filtered CuK_α_ radiation (λ = 0.1544 nm), fixed time mode, step-size of 0.02°, and collecting time of 1.6 s. Structural parameters were calculated by Rietveld’s method, using GSAS software and EXPGUI interface, with crystallographic datasheets from the Ti’s phase (ICSD: α-Ti #43,416 and β-Ti #44,391) and standard Y_2_O_3_ sample for instrumental contribution. Specific details about the refinement quality can be found in Supplementary Material 1. Microstructural characteristics were revealed by optical microscopy (OM; Olympus BX51M microscope) and scanning electron microscopy (SEM; EVO LS15 microscope, Carl Zeiss Inc.). For this, the samples were previously submitted to standard metallographic procedures composed of SiC waterproof paper grinding (#180 to #1500), polishing with alumina (0.25 µm) and silica (0.10 µm) colloidal suspensions, and etching in Kroll’s solution.

Mechanical properties were evaluated by Vickers micro-hardness (HMV-2 hardener, Shimadzu Inc., 0.300 kgf for 15 s), Young’s modulus, and damping factor (excitation impulse method, Sonelastic equipment, ATCP Physical Engineering Inc.). Electrochemical properties were evaluated by open circuit potential (OCP, 3.6 ks), potentiodynamic polarization (PDP, − 1 to 2 V, scan rate of 10 mV s^−1^), and electrochemical impedance spectroscopy (EIS, 10^–1^ to 10^6^ Hz, amplitude of 10 mV, and 10 points per decade) tests. The sample was set up as a working electrode, an Ag/AgCl electrode as a reference, and a Pt disc as a counter electrode. The tests were performed in a simulated marine solution (3.5% NaCl) at room temperature, using a potentiostat (Metrohm Autolab Inc) coupled with an impedance module. The results were analyzed by the NOVA software 2.1 version. Details about the EIS fitting of the data are shown in Supplementary material 2. Tribocorrosion behavior was evaluated in the same conditions as the electrochemical tests, having the sliding of an alumina sphere (diameter of 6 mm) under a load of 1.55 N for 1.8 ks and frequency of 1 Hz. SEM and confocal laser microscopy (DCM3D equipment, Leica Inc.) imaging later analyzed the wear track, which was also used to calculate the average (R_a_) and root mean square (R_rms_) roughness. The tests were taken in triplicate for accurate calculation of the average values. Some results were compared to CP-Ti grade 4, a commonly used material for manufacturing medical implants.

## Results and discussion

**Figure 1:**
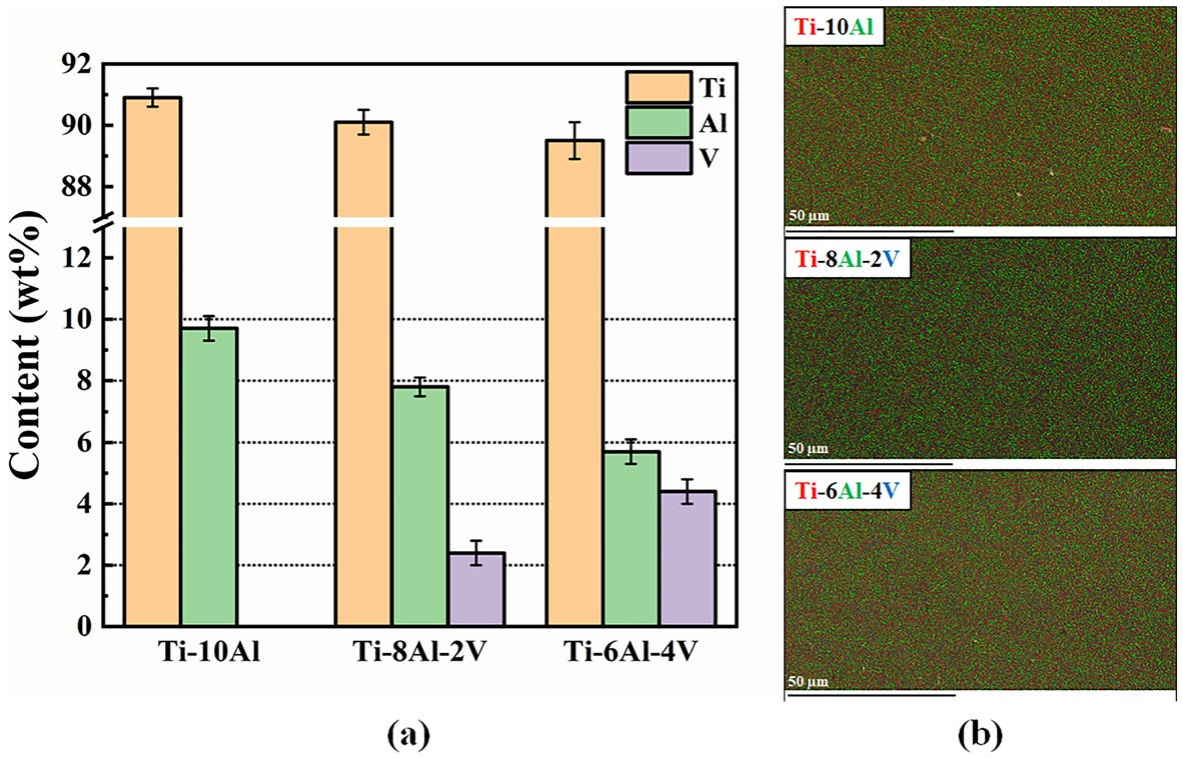
EDS chemical analysis: (**a**) Semi-quantitative results and (**b**) Elemental mapping.

Figure 1 shows the EDS results for the samples after solution heat treatment. The average chemical composition of the samples acquired from three distinct areas (Fig. 1a) magnified at 1000 × remained close to the nominal values (deviation below 1%). The elemental mapping (Fig. 1b) collected in the same magnification depicted a good distribution of the alloying elements without a precise formation of agglomerates at the scale of dozens of micrometers. The results followed the chemical specifications of the ASTM F136 standard^2^ and ensured that the processed samples had good quality for the study.

**Figure 2:**
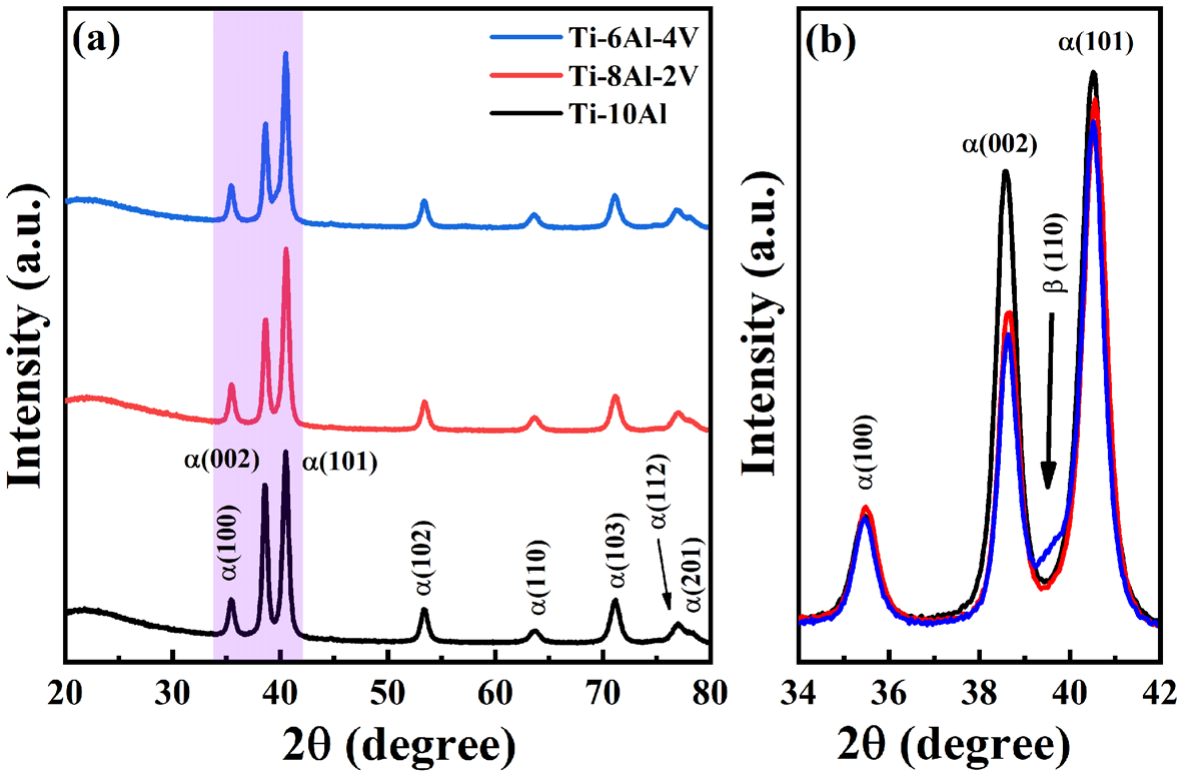
Phase composition analysis: (**a**) extended and (**b**) zoomed XRD profiles.

The phase composition of the samples verified by the XRD results is exhibited in Fig. 2. The XRD patterns (Fig. 2a) of the Ti-(10−x)Al-xV (x = 0, 2, and 4 wt.%) samples indicated diffracted peaks related to the hexagonal close-packed structure (α-Ti phase). Zoom in the region of interest (Fig. 2b) shows the formation of a minor amount of body-centered cubic structure (β-Ti phase) with the V addition, evidenced by the decay on the intensity of (002)_α_ and (101)_α_ peaks and the appearance of a tiny peak around 39.5° related to (110)_β_ peak in the Ti-6Al-4V sample. This result originated from the α- and β-stabilizer action of the Al and V atoms, respectively, which can modify the β-transus temperature of the Ti when used as alloying elements^10^.

**Figure 3:**
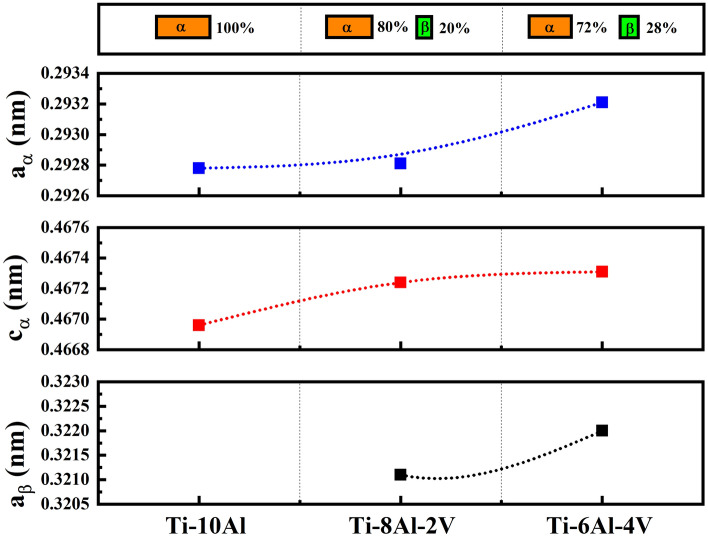
Phase composition and cell parameters.

The phase proportion and cell parameters of the Ti-(10−x)Al-xV (x = 0, 2, and 4 wt.%) samples obtained from the Rietveld refinement are shown in Fig. 3. The results indicate an apparent α → β phase transformation with the addition of V in the solid solution, changing from α- to α + β-type Ti alloy. For example, the Ti-10Al sample displayed a single α phase, with cell parameters significantly lower than the CP-Ti (a_α_ = 0.2951 nm and c_α_ = 0.4684 nm), which can be related to the minor metallic radius of Al (0.143 nm) compared to Ti (0.147 nm)^10,15^. However, the amount of V gradually increased the α and β cell parameters due to its higher metallic radius (0.205 nm)^15,16^. As well as in the α phase, the a_β_ values were also lower than the CP-Ti (0.3311 nm)^10^, indicating that the Al and V atoms were diluted in both phases. Similar results were found by Slokar, Matkovic, and Matkovic^17^ for some Ti–Cr–Nb alloys, whose noted significant variation of the α and β phase cell parameters with the atomic radius of the alloying elements.

**Figure 4:**
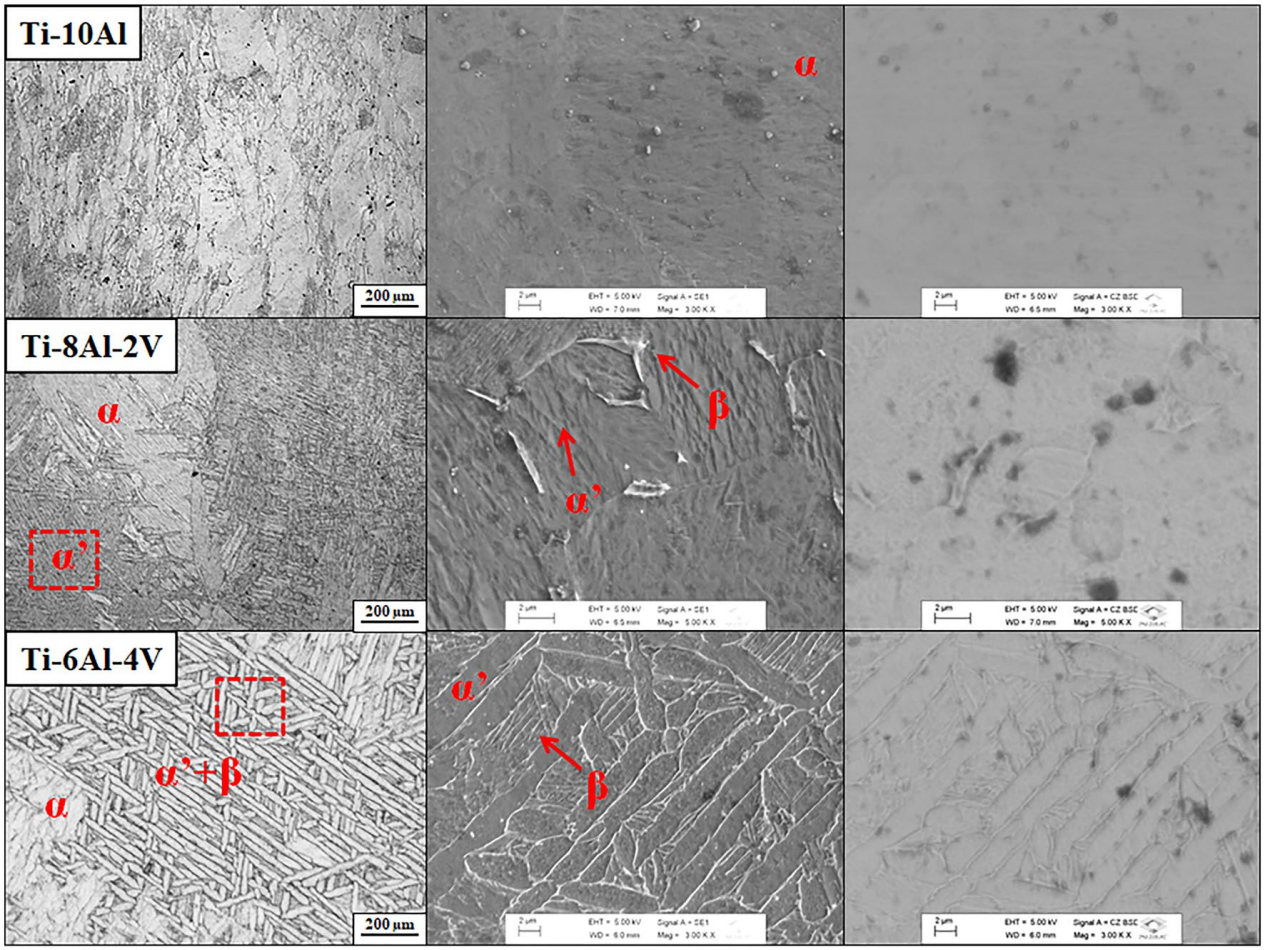
Microstructural analysis: OM (left), SE-SEM (middle), and BSE-SEM (right) imaging.

Microstructural aspects of the Ti-(10−x)Al-xV (x = 0, 2, and 4 wt.%) samples are depicted in Fig. 4, which depicts OM together with SEM images collected using secondary (SE) and backscattered electron (BSE) beams. The red dotted square represents the region of SEM imaging. The Ti-10Al sample was composed of some elongated grains of α phase with dimensions of some hundreds of micrometers. In contrast, the Ti-8Al-2V sample presented plates of α phase permeated by some acicular structures typical of the martensitic α' phase. The SE-SEM imaging of the martensitic region revealed the presence of some irregular precipitates of the β phase. The Ti-6Al-4V sample also displayed lamellae-shaped α phase and a basket wave pattern resulting from α′ + β phases. The corresponding SE-SEM imaging indicated more precipitation of the β phase through the α’ phase. The BSE-SEM images exhibited some dark spots naturally resulting from the metallographic etching process, with some tendency to appear Z-contrast in the α′/β boundary, resulting from the preferential location of the alloying elements with different atomic numbers (Ti = 22, Al = 13, and V = 23)^15^. It is well known that the combination of β-stabilizer elements with proper heat treatment can induce the precipitation of metastable phases, such as the martensitic α’ phase, which is formed with a low quantity of alloying elements^18^. As the solutionized samples suffered water quenching from temperatures above the β-transus, it provoked β → α′ phase transformation together with the natural β → α during the cooling. The martensitic α′ phase has a distorted hexagonal-close packed crystal structure with the same spatial group of α-Ti^19^. Thus, this phase was impossible to be distinguished by using conventional XRD measurements.

**Figure 5:**
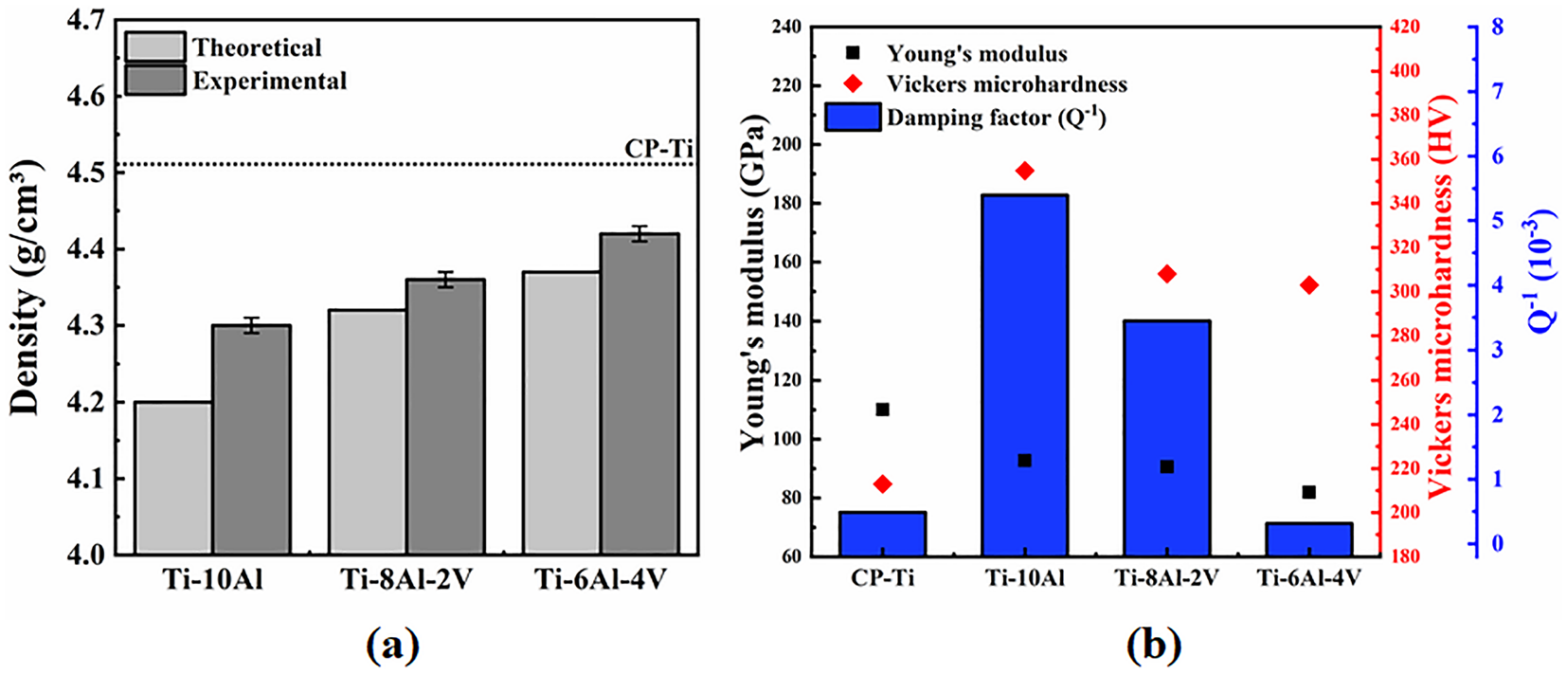
Selected properties: (**a**) density and (**b**) mechanical values.

The samples' density and mechanical properties values compared to CP-Ti for Ti-(10-x)Al-xV (x = 0, 2, and 4 wt.%) samples are shown in Fig. 5. The experimental density values (Fig. 5a) exhibited the same trend as the theoretical values, gradually increasing with the V addition. The increment in the density values is produced by the higher density value of V (6.11 g cm^-3^) when compared to Ti (4.51 g cm^−3^) and Al (2.70 g cm^−3^)^15^, even though all the samples presented values below the CP-Ti. From the amputee’s point of view, light materials can be an advantage for manufacturing knee prostheses once it could result in minor efforts to get mobility^1^. The selected mechanical properties of the samples are compared to the CP-Ti as depicted in Fig. 5b. The Young’s modulus remained below the CP-Ti, showing step decay with the amount of V, provided by the precipitation of metastable α′ and β phases^10^. As earlier reported, materials with low Young’s modulus, close to the human cortical bones (~ 30 GPa), can supply proper transmission of biomechanical loads, avoiding bone atrophy caused by the stress shielding effect^5^. Contrarily, the Vickers micro-hardness values were higher than CP-Ti, resulting in solid solution and phase precipitation hardening mechanisms provoked by the alloying elements and the metastable phases^18,20^. The slight decrease of micro-hardness with the amount of V resulted in less hardener than Ti in solid solution^21^. Hardness is directly related to mechanical strength, so hard metallic materials can exhibit favorable mechanical properties for prostheses. Regarding the damping factor (Q^−1^), the Ti-10Al sample exhibited a higher value than the samples, highlighting its ability to absorb mechanical vibrations without significant deformation. High-damping materials can provide better support for mechanical loads without failure, which could be helpful for load-bearing biomedical materials^22^. From the mechanical point of view, the potential applicability for use as knee prostheses can be listed as Ti-10Al > Ti-8Al-2V > Ti-6Al-4V > CP-Ti.

**Figure 6:**
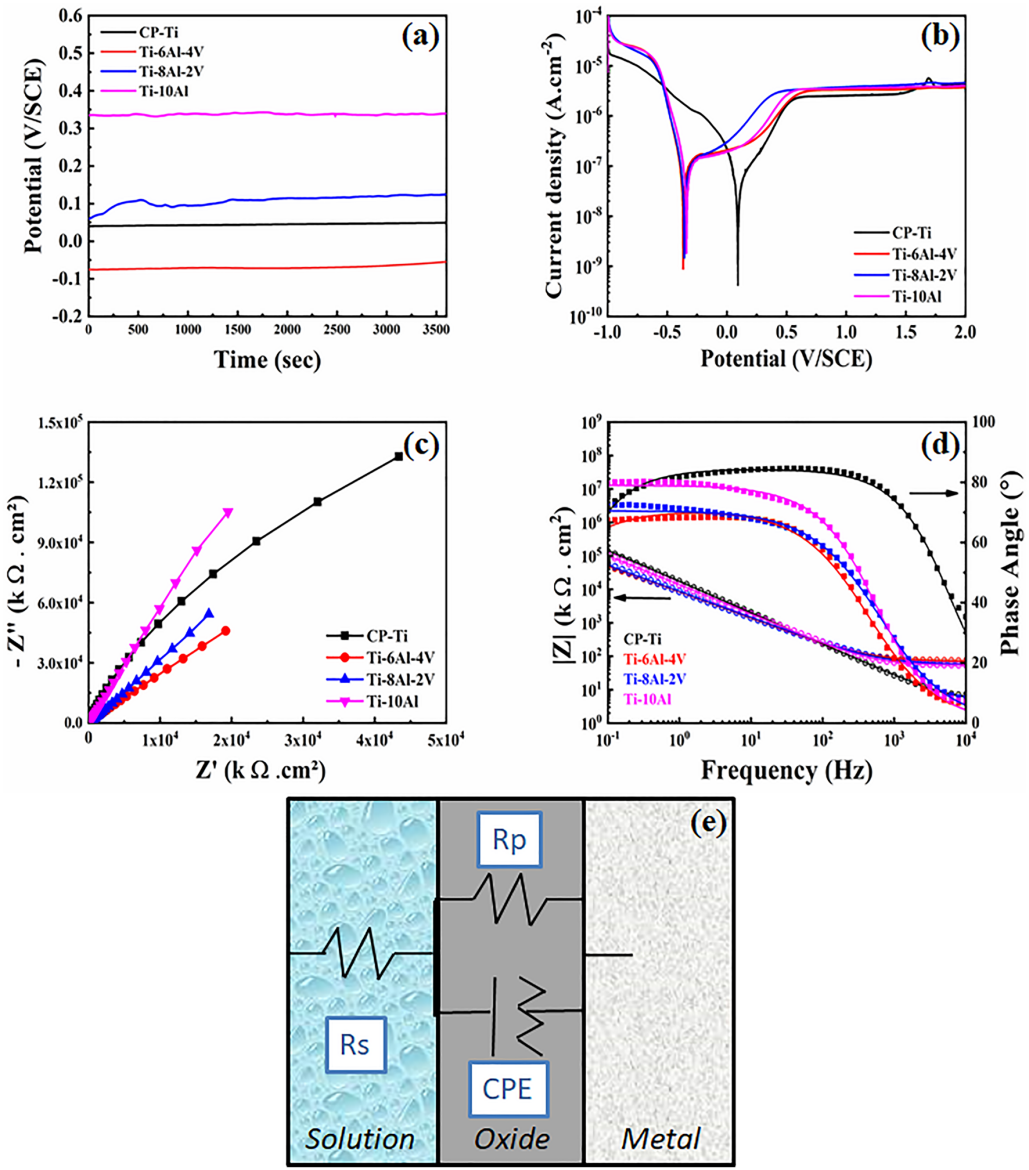
Electrochemical analysis: (**a**) OCP, (**b**) PDP, (c) and (**d**) EIS results, and (**e**) equivalent electric circuit.

The results obtained from the electrochemical tests conducted in simulated marine solution (3.5% NaCl) for Ti-(10−x)Al-xV (x = 0, 2, and 4 wt.%) samples are shown in Fig. 6. The OCP (Fig. 6a) values of the Ti-10Al sample had the noblest behavior, indicating a more stable passive oxide layer on the surface. The PDP (Fig. 6b and Table 1) results indicate that all samples possessed a corrosion potential (E_corr_) below the CP-Ti with a slightly higher corrosion current (j_corr_), which could indicate minor resistance to polarization and degradation of the surface. However, the polarization resistance (R_p_) and corrosion rate (C_R_) values remained at the same magnitude, indicating similar behavior against the simulated marine solution. In addition, considering that the Ti-6Al-4V sample is already commercially used as a biomedical metallic material, the other samples exhibited suitable results for the application as knee prostheses. Furthermore, in the cathodic region at higher potentials, CP-Ti was more susceptible to pitting due to the typical oscillations after the passive layer breaking, while the other samples remained stabilized. The Nyquist plot from the EIS results is shown in Fig. 6c, where an apparent single semi-circle can be seen in all samples. The diameter of the semi-circle for the Ti-10Al sample was the largest, indicating a probable higher polarization resistance than the other samples^23^. The equivalent electrical circuit in Fig. 6e shows a combination of a resistive component from the solution and a single parallel electrical circuit from the oxide layer, denoted by a Randles circuit. The circuit comprised the polarization resistance of the solution (R_s_) and oxide layer (R_p_), and a constant phase element (CPE). Chávez-Díaz et al.^24^ reported that the passive layer of the Ti-6Al-4V alloy is composed mainly of TiO_2_ and its suboxides (TiO and Ti_2_O_3_), and a minor amount of Al_2_O_3_, which positively contributed to increasing the corrosion resistance in Hank’s solution. From the EIS fitted parameters indicated in Table 2, it is possible to observe that the samples possessed higher polarization resistance value of their oxides (R_p_) compared to the CP-Ti, indicating significant protection against the simulated marine environment. The Bode diagram (Fig. 6d) shows that CP-Ti at low frequency has similar resistance to the other samples, which means an excellent behavior of the protective layer of TiO_2_. On the other hand, at high frequency, CP-Ti presented the highest capacitance among the samples, allowing a significant passage of electrons through the surface and the occurrence of degradation mechanisms. The phase constant exponent α denotes de electrical behavior of the CPE component, being all results obtained from the oxides were closer to the 1.0 value and indicated a capacitive characteristic. Still, the Ti–Al–V samples exhibited minor ones, indicating a tendency for resistive behavior. As it is known, a capacitive behavior indicates that ordered charges on the surface are permissible for electrons to pass through it. In contrast, the resistive behavior forms a protective layer that does not allow the passage of electrons^25^. The material’s corrosion resistance against simulated marine solutions permits the usage of prostheses in seawater environments^26^, increasing the life quality and integration of the amputee in society. From the electrochemical point of view, the samples can be classified as Ti-10Al > CP-Ti > Ti-8Al-2V > Ti-6Al-4V.

**Table 1:** Tafel parameters obtained from the PDP curves.

	CP-Ti	Ti-10Al	Ti-8Al-2V	Ti-6Al-4V
E_corr_ (V)	0.09	− 0.34	− 0.35	− 0.37
j_corr_ (A.cm^-2^)	5.2 × 10^–8^	5.9 × 10^–7^	1.3 × 10^–7^	8.6 × 10^–7^
R_p_ (Ω)	6.0 × 10^5^	3.6 × 10^5^	3.4 × 10^5^	3.8 × 10^5^
CR (mm/year)	0.1 × 10^–5^	2.3 × 10^–5^	0.5 × 10^–5^	0.3 × 10^–5^

**Table 2:** EIS parameters obtained from the equivalent electric circuits.

	CP-Ti	Ti-10Al	Ti-8Al-2V	Ti-6Al-4V
R_s_ (Ω)	5.05	53.95	55.77	69.71
R_p_ (Ω)	5.76 × 10^5^	1.10 × 10^12^	1.10 × 10^12^	4.56 × 10^5^
CPE (C)	1.01 × 10^–5^	1.46 × 10^–5^	2.70 × 10^–5^	2.76 × 10^–5^
α	0.94	0.88	0.78	0.79

**Figure 7:**
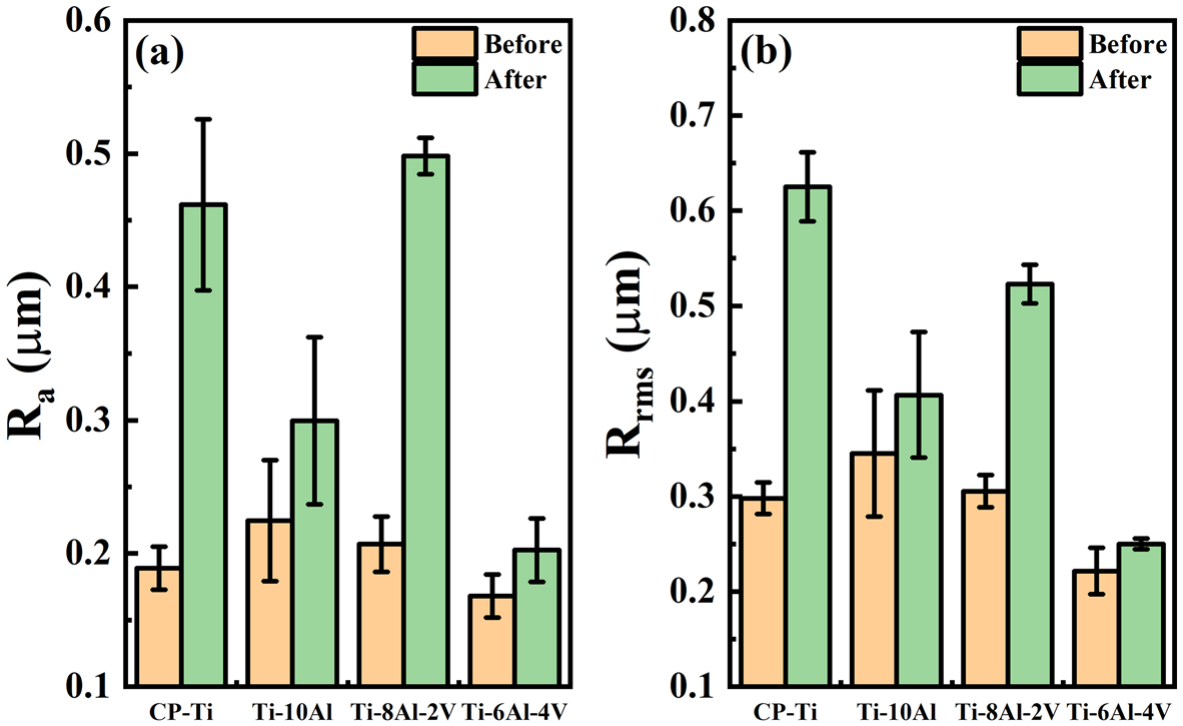
Roughness changes with the electrochemical analysis: (**a**) R_a_ and (**b**) R_rms_ values.

The roughness values of the Ti-(10−x)Al-xV (x = 0, 2, and 4 wt.%) samples taken before and after the electrochemical tests are compared in Fig. 7. It is possible to note that the corrosive mechanisms that occurred on the surface resulted in significant changes in the R_a_ e R_rms_ values in all samples. Rough surfaces can indicate the presence of pitting corrosion and more tendency to corrosion^27^. Chi, Yi, and Liu^28^ found a significant effect of roughness on the electrochemical properties and pitting corrosion in the Ti-6Al-4V alloy in an acidic HCl-based solution once the irregular topography somehow delays the oxide passivation of the surface. In this sense, the Ti-10Al and Ti-6Al-4V samples, which exhibited minor variations in the roughness values, depicted the most favorable results.

**Figure 8:**
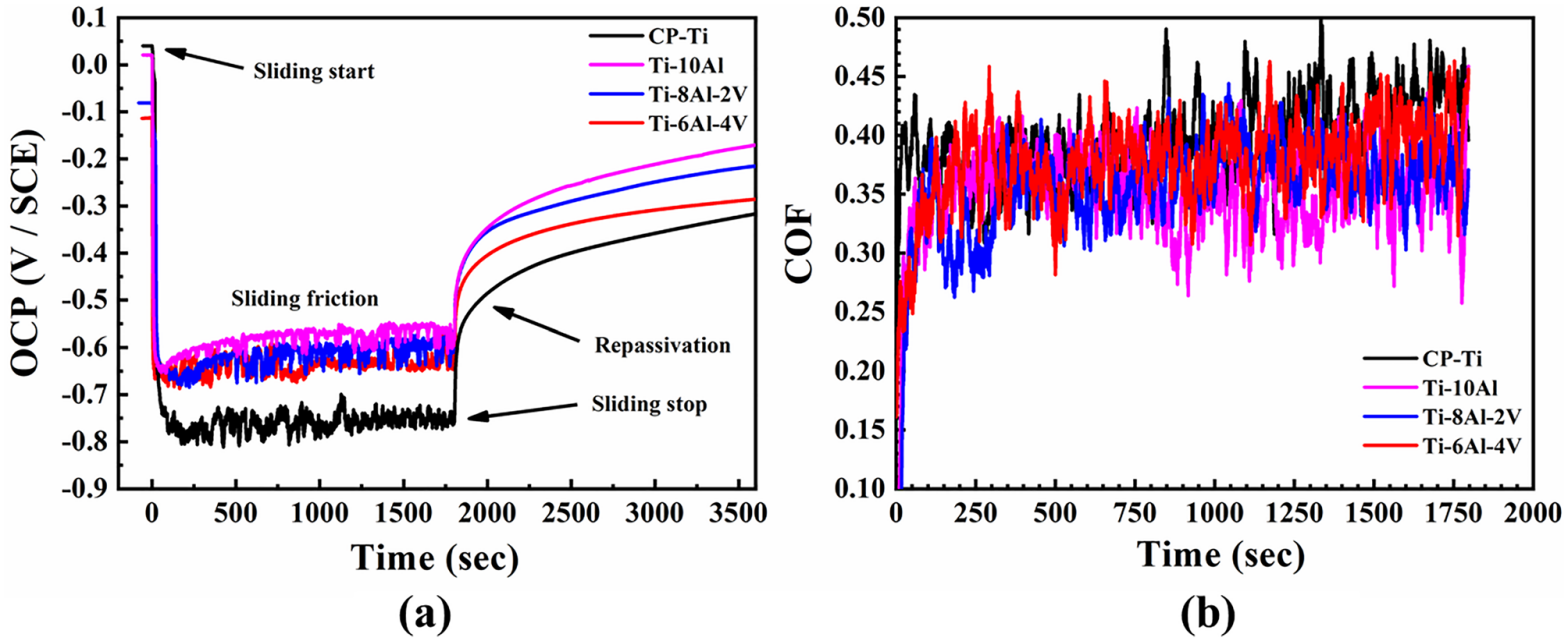
Tribocorrosion analysis: (**a**) OCP and (**b**) COF results.

The OCP values of the Ti-(10−x)Al-xV (x = 0, 2, and 4 wt.%) samples recorded during the tribocorrosion test and the corresponding COF values are shown in Fig. 8. Overall, the results followed the typical characteristics of biomedical Ti alloys when submitted to wear and corrosion mechanisms in aqueous corrosive environments^29^. During the sliding friction (Fig. 8a), the OCP values decayed sharply for all samples due to the removal of the passive oxide layer by the counter body (depassivation). Regarding the initial and final OCP values, CP-Ti showed a more significant gap, indicating that the samples produced were nobler due to their more positive OCP to CP-Ti during sliding. The rugged curves were higher in CP-Ti, resulting from the constant removal and repassivation of the oxide layer and the deposition of tribolayers on the surface from the debris. However, as this debris can also be released into the human body, they had a noticeable impact on cytokines production, which can decay cell viability^30^. The Ti-10Al sample exhibited the noblest OCP values, indicating better surface stability during the sliding. With the sliding stopping, all samples depicted better repassivation ability than the CP-Ti, as indicated by the higher OCP values. Although the most stable after repassivation was the Ti-6Al-4V because the line is almost straight, the Ti-10Al had the best tendency to repassivation on return when compared to all the studied alloys. Despite this, all samples' COF values remained almost the same (around 0.30–0.40). Thus, from the tribocorrosion point of view, the samples can be classified as Ti-10Al > Ti-8Al-2V > Ti-6Al-4V > CP-Ti.

**Figure 9:**
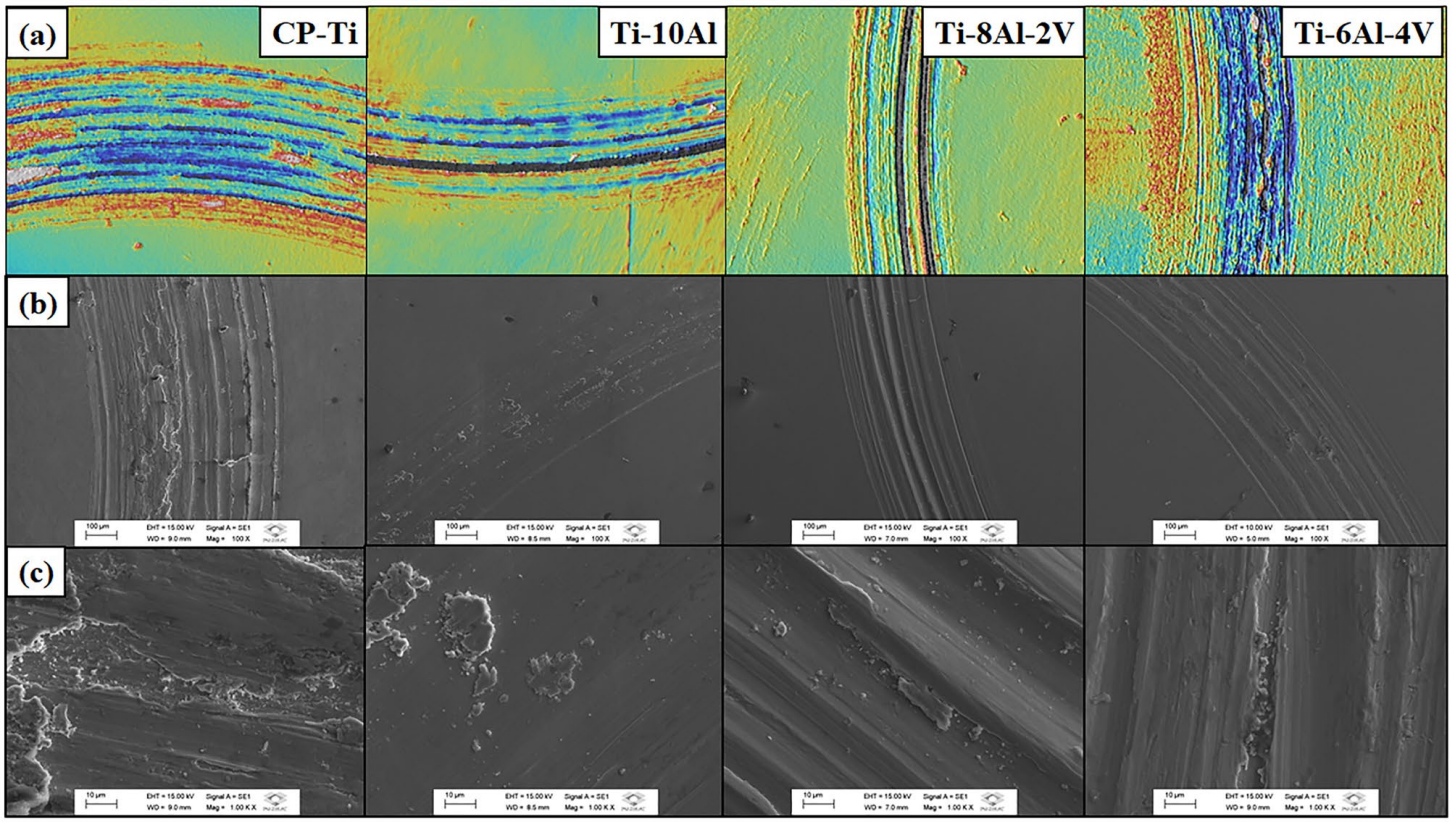
Topography of the wear tracks: (**a**) confocal 3D laser imaging at 10x and SE-SEM imaging at (**b**) 100x and (c) 1000x.

The confocal 3D laser imaging for Ti-(10−x)Al-xV (x = 0, 2, and 4 wt.%) samples are shown in Fig. 9. It is possible to evaluate the topography of the wear track resulting after the tribocorrosion test. The width of the wear track of the samples remained lower than the CP-Ti, indicating better tribocorrosion resistance and minor wear volume. All the wear tracks presented typical grooves resulting in abrasive wear with the counter body^31^. The SEM images (Fig. 9b and c) show that the wear track of the Ti-10Al sample seemed flatter with some amount of deposited tribolayer plates originating from the deposition of wear debris, which is typical of adhesive wear. The samples depicted a wear track with a width of around 300 µm while the CP-Ti presented 500 µm. Feyzi et al.^32^ reported the same wear mechanisms and investigated the effect of normal force and the applied potential on the tribocorrosion behavior of the Ti-6Al-4V alloy in PBS (Phosphate-Buffered Saline) solution.

**Figure 10:**
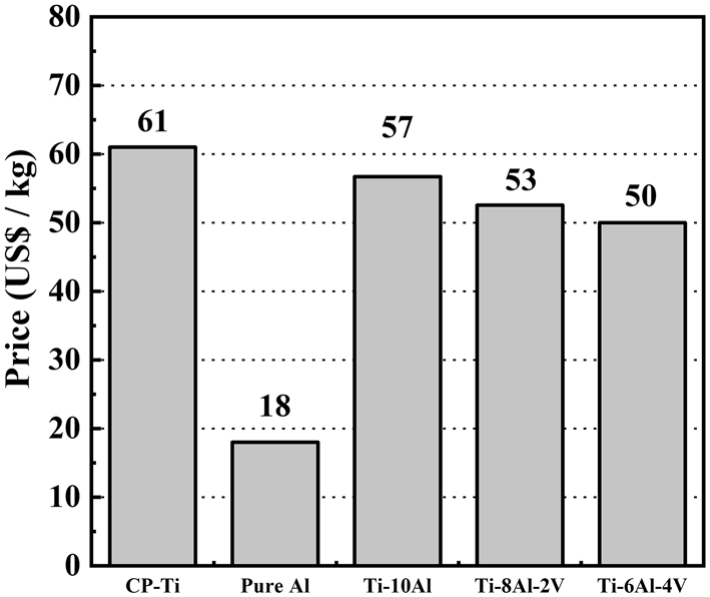
Estimated price to manufacture the samples.

Figure 10 shows an estimate of the price to manufacture the material based only on the market pricing of the raw materials. It compared the average world price (US$ per kg) for the CP-Ti grade 2, pure Al, and Ti-6Al-4V reported by the Berkeley Lab (www.materialslab.org). The estimated price of the Ti-(10−x)Al-xV (x = 0, 2, and 4 wt.%) alloys was calculated from the weighted values of the raw materials used in this study. Pure Al has elevated recyclability and the Ti-6Al-4V alloy is primarily marketed worldwide, so their pricing is lower than the CP-Ti. As a result, using these materials as alloying elements provided more attractive price values for the Ti-10Al and Ti-8Al-2V alloys. Thus, considering the mechanical, electrochemical, and tribocorrosion properties, the Ti-10Al alloy could be the best choice for manufacturing single-axis knee prostheses, ensuring low cost and usage for the long term.

## Conclusions

Ti-(10−x)Al-xV (x = 0, 2, and 4 wt%) samples were produced and characterized for potential application as single-axis knee prostheses. The samples exhibited an excellent mixture of the alloying elements on the scale of dozens of micrometers, having a primary α phase with minor amounts of metastable α' and β phase induced by the β-stabilizer V alloying in the solid solution. The cell parameters changed with the chemical composition, depending on the atomic radius of the substitutional Al and V elements. The microstructure was initially composed of α phase elongated grains, changing from a basketweave structure typical of dual α′ + β phase. Due to the chemical and phase composition, the Vickers microhardness, Young’s modulus, and damping factor changed gradually. The electrochemical tests indicated adequate corrosion resistance against the simulated marine solution (3.5% NaCl). The tribocorrosion tests also depicted exciting results for the application, being the wear track evidence of abrasive and adhesive wear mechanisms. The Ti-10Al samples exhibited the best mechanical, electrochemical, and tribocorrosive properties for usage as a single-axis knee component, especially attractive price concerning CP-Ti grade 2, giving new horizons for novel developments of Ti-based alloys targeted for an external prosthesis. Further research on the advanced manufacturing of porous Ti-10Al, such as by using 3D printing technologies, can benefit in reducing the weight and costs of the prosthesis without significantly impairment of these properties.

## Supplementary Information

Supplementary Information 1.

Supplementary Information 2.

## Data Availability

The data can be shared under request to the e-mail address: diego.correa@unesp.br.
