# Supplementary material for: Development of Ti–Al–V alloys for usage as single-axis knee prostheses: evaluation of mechanical, corrosion, and tribocorrosion behaviors
Source: Sci Rep. 2023 Mar 16;13:4349. doi: 10.1038/s41598-023-31548-1 (PMC10020473; doi:10.1038/s41598-023-31548-1)
Supplement: Supplementary file 1 — Supplementary Information 1. [file 41598_2023_31548_MOESM1_ESM.docx]

**Supplementary Material 1 - Rietveld refinement details**

**Table S1-1 –** Merit parameters.

| **Sample** | **χ^2^** | **Rwp (%)** | **Rp (%)** | **RF² (%)** |
| --- | --- | --- | --- | --- |
| **Ti-10Al** | 2.191 | 4.18 | 2.99 | 2.43 |
| **Ti-8Al-2V** | 2.741 | 4.85 | 3.80 | 3.61 |
| **Ti-6Al-4V** | 2.810 | 5.07 | 3.72 | 3.23 |

|  |
| --- |
|  |
|  |

**Figure S1-1 –** Refinement plots.
