# Supplementary material for: Development of Ti–Al–V alloys for usage as single-axis knee prostheses: evaluation of mechanical, corrosion, and tribocorrosion behaviors
Source: Sci Rep. 2023 Mar 16;13:4349. doi: 10.1038/s41598-023-31548-1 (PMC10020473; doi:10.1038/s41598-023-31548-1)
Supplement: Supplementary file 2 — Supplementary Information 2. [file 41598_2023_31548_MOESM2_ESM.docx]

**Supplementary Material 2 – EIS fitting details**

| 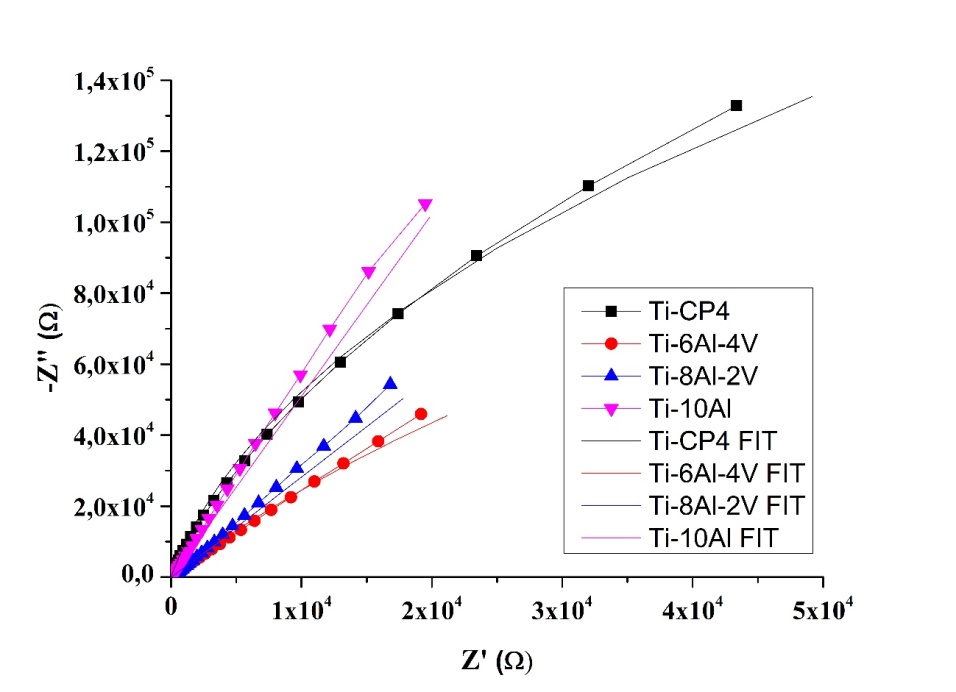  (a) |
| --- |
| 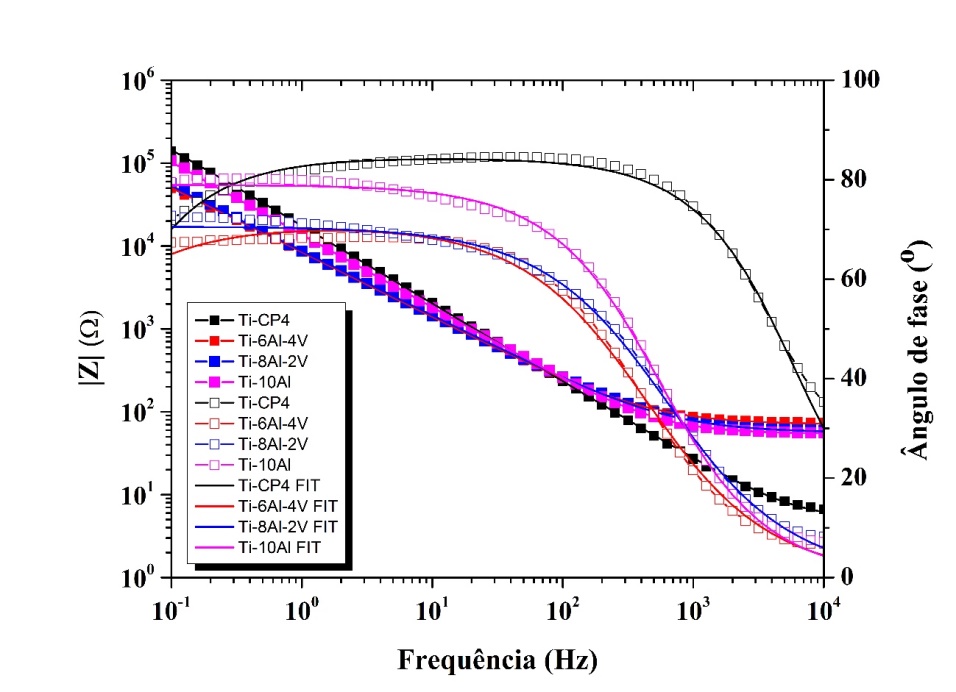  (b) |

**Fig. S2-1 –** EIS fitting plots of the samples: (a) Nyquist and (b) Bode curves.

**Table S2-1 -** Equivalent electric circuits.

| **Ti-CP4** | 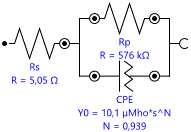 |
| --- | --- |
| **Ti6Al4V** | 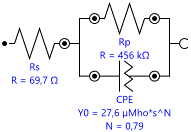 |
| **Ti8Al2V** | 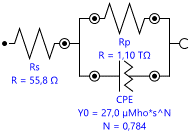 |
| **Ti10Al** | 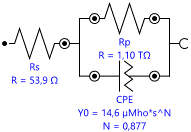 |

**Table S2-2 -** EIS fitted parameters from the equivalent electric circuit.

|  | **CP-Ti** | **Ti-10Al** | **Ti-8Al-2V** | **Ti-6A-4V** |
| --- | --- | --- | --- | --- |
| **R_s_ (Ω)** | 5.05 | 53.95 | 55.77 | 69.71 |
| **R_p_ (Ω)** | 5.76 x 10^5^ | 1.10 x 10^12^ | 1.10 x 10^12^ | 4.56 x 10^5^ |
| **CPE (C)** | 1.01 x 10^-5^ | 1.46 x 10^-5^ | 2.70 x 10^-5^ | 2.76 x 10^-5^ |
| **α** | 0.94 | 0.88 | 0.78 | 0.79 |
| **χ²** | 0.05 | 0.04 | 0.03 | 0.03 |

## **Symbols caption:**

- R_s_: Solution Resistance [Ω]
- R_p_: Polarization Resistance [Ω]
- CPE: Constant Phase Elements (Q)
- α: Charge transfer coefficient (used to define kinetics of electrochemical reaction)
- χ²: Chi-square value (for better the fitting, it should remain closer to zero)
